# Reliability and Factorial Structure of the Farsi Version of the Arabic Scale of Death Anxiety in an Iranian Middle-Aged Sample

**DOI:** 10.1155/2016/9457041

**Published:** 2016-11-28

**Authors:** Mahboubeh Dadfar, Fazel Bahrami

**Affiliations:** ^1^School of Behavioral Sciences and Mental Health-Tehran Institute of Psychiatry, International Campus, Iran University of Medical Sciences, Tehran, Iran; ^2^Department of Islamic Theology and Department of Counseling, University of Social Welfare and Rehabilitation Sciences, Tehran, Iran

## Abstract

The present study aimed to explore the psychometric properties of the Arabic Scale of Death Anxiety (ASDA) in an Iranian middle-aged sample. A sample of 55 volunteer Iranian persons took part in the study. Cronbach's alpha of the ASDA was found to be high (0.91) and Spearman-Brown and Guttman Split-Half coefficients were 0.86. The factor analysis of the ASDA items yielded five factors accounting for 72.49% of the total variance and labeled (F1) fear of death and fear of dead people; (F2) fear of postmortem events and fear of tombs; (F3) fear of lethal disease; (F4) preoccupation with after death, and death fear in sleep; and (F5) fear of deprivation of own ones. The ASDA has a good validity and reliability, and it can be used in clinical, educational, and research settings.

## 1. Introduction

Death anxiety is one of psychological important components. People experience varying degrees of anxiety death in their life. Individuals conceal their death anxiety in their own groups. Despite the denial, they experience psychological stress and symptoms of death anxiety [1]. Age operates as a significant factor in death anxiety [2].

Fortner and Neimeyer indicated that death anxiety is at its highest level in the middle age, then decreases in late middle age, and remains in the elderly [3].

Cicirelli found middle-aged persons more experience death anxiety than elders because they feel dissonancy between their desires with spent expected time in their lives [4].

There is a significant difference in the rate of awareness consciousness of death anxiety in developmentally different ages. The construct of midlife crisis as a developmental event occurs in middle-aged persons. Middle-aged individuals encounter one's own death or death of others. Death is one phenomenon which can exacerbate midlife crisis anxiety. Death anxiety or death fear is one of common causes of midlife crises. The anxious personality patterns of types complicate midlife crisis [5–8].

One of the correlates influencing death anxiety is age [9–18]. Death anxiety affects health promoting behaviors and somatic health in young adults [19, 20].

The Arabic Scale of Death Anxiety (ASDA) is one of the scales for assessing death anxiety [21]. It has been used in many samples [22–25].

Some studies related to death anxiety have been done on Iranian different samples such as college students and nurses using various tools, for example, Templer Death Anxiety Scale and Collet-Lester Death Fear Scale. The findings showed that rate of death anxiety is high in the samples. The aim of present study is to explore the psychometric properties of the Farsi form of the ASDA among Iranian middle-aged sample who lives in Tehran. Tehran is Iran*'*s capital city and has a population density of 11,887 people. Tehran has the most middle-aged and old-aged population in the country.

## 2. Methods

### 2.1. Participants

The study was a cross-sectional, descriptive study. A sample of 55 volunteer Iranian middle-aged individuals took part in the study. They were selected by a convenient sampling. The middle-aged persons had come to the neighborhood parks for leisure, recreation, and the activities done for enjoyment when they were not working. The study is limited to the middle-aged persons located at Tehran city. The mean ages were 52.63 (SD = 4.52), and the age range was from 50 to 56, for men 52.25 (SD = 4.70) and for women 55.28 (SD = 1.38), respectively. 85.7% were male and 14.3% female. Although the sample was nonrandom but due to more presence of men in the neighborhood parks, they were more than the women. The subjects more often had a diploma's degree (53.2%) and were married (91.8%).

### 2.2. Measures

Demographic characteristics of the sample include age and gender. The Arabic Scale of Death Anxiety (ASDA) was used. The ASDA was developed by Abdel-Khalek and validated in Egypt, Kuwait, Syria [21], Turkey, and Iran countries [10]. It has 20 items, and each item is answered on a 5-point intensity scale anchored by 1 (no) and 5 (very much). Good validity with other scales and reliability by Cronbach's alpha and test-retest methods have been reported for the ASDA [22–24]. In the present study, Farsi version of the ASDA was used.

## 3. Results

The mean score of the ASDA was 43.94 (SD = 14.90), men 42.16 (SD = 14.63), and women 50.28 (SD = 17.32), respectively. Middle-aged men had higher mean ASDA total score than women. This difference was not significant. The lowest mean score was 1.25 (SD = .72) for item (3); and the highest mean score was 3.12 (SD = 1.55) for item (6) (Table 1).

### 3.1. Reliability of the ASDA

For the middle-aged individuals' sample, the Cronbach alpha was 0.91, the Spearman-Brown coefficient was 0.86, and the Guttman coefficient was 0.86. The intercorrelations between the items ranged from 0.007 (for items (10) and (13)) to 0.752 (for items (2) and (16)), and the item-total correlations ranged from 0.380 (for item (13) and total score) to 0.800 (for item (18) and total score) in the sample.

### 3.2. Factor Analysis of the ASDA

The criteria for the factor analysis were evaluated using the Kaiser-Meyer-Olkin Measure of Sampling Adequacy (KMO) and the Bartlett Test of Sphericity. The KMO was 0.803, indicating the adequacy of the sample of college students, and Bartlett's Test of Sphericity was 715.148 (df = 190, *p* < .001) indicating that the factor analysis was justified in the middle-aged sample.

Five components with eigenvalues greater than one were retained in the sample of the middle-aged as reported in Table 2. Inspection of this table reveals that Factor 1 (9 items) explained 40.53% of the observed variance and was labeled “Fear of death and fear of dead people.” It included the items: “I fear looking at the dead,” “I fear visiting graves,” “I am afraid of looking at a corpse,” “Witnessing the burial procedure terrifies me,” “I dread walking in graveyards,” “I get upset by witnessing a funeral,” “The sight of a dying person frightens me,” “Talking about death upsets me,” and “I fear death.”

Factor 2 (5 items) explained 12. 37% of the observed variance and was labeled “Fear of postmortem events and fear of tombs” and included the items: “I am apprehensive of unknown things after death,” “I fear the torture of the grave,” “I fear getting a serious disease,” “The pain accompanying death terrifies me,” and “I am afraid of getting cancer.”

Factor 3 (3 items) explained 7.79% of the observed variance and was labeled “Fear of lethal disease.” It included the items: “I fear death whenever I because ill,” “The possibility of having a surgical operation terrifies me,” and “I am afraid of suffering heart attack.”

**Figure 1:**
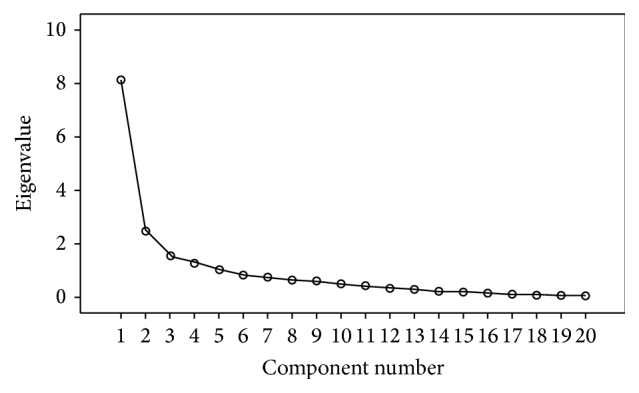
Scree plot of the ASDA.

The remaining factors had one or two items. Factor 4 (2 items) explained 6.53% of the observed variance and was labeled “Preoccupation with after death, and death fear in sleep.” It included the items: “I am preoccupied with thinking about what will happen after death” and “I am afraid of sleeping and not waking up again.” Factor 5 (1 item) explained 5.25% of the observed variance and was labeled “Fear of deprivation of own ones.” It included the items: “I worry that death deprives me of someone dear to me” (Tables 2 and 3 and Figure 1).

## 4. Discussion and Conclusion

The results indicate that mean score of the ASDA was 43.94 (SD = 14.90). The means for the ASDA were rated in the range of “no” to “very much.” The ASDA items receiving the lowest mean score in the present sample of middle-aged persons were as follows: item (3) “I fear visiting graves” (1.25); and the highest mean score item (6) “I worry that death deprives me of someone dear to me (3.12), item (9) “I fear the torture of the grave” (3.10), and item (10) “I fear getting a serious disease” (3.03). The intercorrelations between the items ranged from low to moderate, and the item-total correlations ranged from moderate to high (significant at the 0.01 level).

The ASDA has high internal consistency, Split-Half, and Spearman-Brown reliabilities (ranging from 0.86 to 0.91). Our findings are similar to previous studies [21–24]. Therefore, we conclude that the ASDA is useful for assessing death anxiety in the Iranian middle-aged, clinical and nonclinical samples to evaluate attitudes toward death and dying in Iranian society.

The principal components analysis identified five components (accounting for 72.49% of the total variance) of the ASDA as follows: “Fear of death and fear of dead people”; “Fear of postmortem events and fear of tombs”; “Fear of lethal disease”; “Preoccupation with after death, and death fear in sleep”; and “Fear of deprivation of own ones.” These factors were replicable with previous factors extracted from a Turkish college students sample [24] and were not similar to Kuwaiti college student sample and a Kuwaiti middle-aged sample [21, 22]. There were some differences in specific factors between the previous results and the present findings. Due to using the ASDA among college students samples with different cultures, various rotation methods, and factor loading coefficients in previous studies, our findings lead to no similar results (except study of Sariçiçek Aydoğan et al.) [24].

The present study has some limitations. Despite the good psychometric characteristics of the ASDA, the selection of the sample was not random, but rather one of conveniences. One of limitations of our study was Tehran middle-aged population in Iran for this scale. There was an overrepresentation of middle-aged males. Future research should study the ASDA in middle-aged females separately from middle-aged males. One of the correlates of death anxiety is religiosity-spirituality. Some studies showed there were associations between concepts related to religiosity and spirituality and death fear and death anxiety [26–32] and the activation of death-related semantic concepts in adults [33]. We did not assess these concepts in our study.

Future research should be addressed to construct validity of the ASDA with the measures related to death, longitudinal and qualitative studies on the middle-aged individuals, investigation of death anxiety and factorial structure of the ASDA on the Iranian public population with various subcultures, and the socio-demographic-psycho-religious, spiritual, and semantic correlates of death anxiety in future studies. However, it is hoped that this study will stimulate further cross-cultural research on the ASDA in the middle-aged individuals.

## Figures and Tables

**Table 1:** Mean and SD of the ASDA items and total scores.

ASDA items	Minimum	Maximum	Mean	SD
(1)	1.00	5.00	1.96	1.10
(2)	1.00	5.00	1.90	1.17
(3)	1.00	5.00	1.25	.72
(4)	1.00	5.00	2.18	1.23
(5)	1.00	5.00	2.54	1.18
(6)	1.00	5.00	3.12	1.55
(7)	1.00	5.00	2.58	1.52
(8)	1.00	5.00	1.85	1.12
(9)	1.00	5.00	3.10	1.57
(10)	1.00	5.00	3.03	1.27
(11)	1.00	5.00	1.85	1.20
(12)	1.00	5.00	1.49	.90
(13)	1.00	5.00	2.21	1.07
(14)	1.00	5.00	1.87	1.09
(15)	1.00	5.00	2.76	1.09
(16)	1.00	5.00	1.69	1.09
(17)	1.00	5.00	1.96	1.09
(18)	1.00	5.00	1.63	1.09
(19)	1.00	5.00	2.81	1.09
(20)	1.00	5.00	2.07	1.09

Total score	23.00	98.00	43.94	14.90

**Table 2:** Factor loadings of the Arabic Scale of Death Anxiety (ASDA) in Iranian middle-aged population (*N* = 55).

(ASDA) items	Component				
	1	2	3	4	5
(1) I fear death whenever I because ill.			.655		
(2) I fear looking at the dead.	.867				
(3) I fear visiting graves.	.670				
(4) The possibility of having a surgical operation terrifies me.			.829		
(5) I am afraid of suffering heart attack.			.721		
(6) I worry that death deprives me of someone dear to me.					.908
(7) I am apprehensive of unknown things after death.		.622			
(8) I am afraid of looking at a corpse.	.803				
(9) I fear the torture of the grave.		.547			
(10) I fear getting a serious disease.		.824			
(11) Witnessing the burial procedure terrifies me.	.837				
(12) I dread walking in graveyards.	.834				
(13) I am preoccupied with thinking about what will happen after death.				.812	
(14) I am afraid of sleeping and not waking up again.				.712	
(15) The pain accompanying death terrifies me.		.707			
(16) I get upset by witnessing a funeral.	.846				
(17) The sight of a dying person frightens me.	.644				
(18) Talking about death upsets me.	.797				
(19) I am afraid of getting cancer.		.857			
(20) I fear death.	.505				
Eigenvalue	8.10	2.47	1.56	1.30	1.05
% of variance	40.53	12.37	7.79	6.53	5.25
% of total variance	72.49%				

Factor 1 (items: 2, 3, 8, 11, 12, 16, 17, 18, and 20): Fear of death and fear of dead people.

Factor 2 (items: 7, 9, 10, 15, and 19): Fear of postmortem events and fear of tombs.

Factor 3 (items: 1, 4, and 5): Fear of lethal disease.

Factor 4 (items: 13 and 14): Preoccupation with after death, and death fear in sleep.

Factor 5 (items: 6): Fear of deprivation of own ones.

**Table 3:** Component transformation matrix of the ASDA.

Component	1	2	3	4	5
(1)	.755				
(2)	−.603	.721			
(3)				.940	
(4)			.861		
(5)					.932

Extraction method: principal component analysis.

Rotation method: Varimax with Kaiser Normalization.
